# Remembering Yoshifumi Ninomiya

**DOI:** 10.1186/1477-5751-13-7

**Published:** 2014-04-23

**Authors:** Bjorn R Olsen

**Affiliations:** 1 Harvard School of Dental Medicine, 188 Longwood Avenue, Boston, MA 02115, USA

## 

It is with great sadness that I sit down at my desk to write about Yoshifumi (Yoshi) Ninomiya, but when I see him in my mind’s eye, the view is filled with sunshine. I believe this is the light in which he wanted us to see him: Someone who was as excited about research in matrix biology when he was close to retirement as when he was a postdoctoral fellow; the professor of biochemistry who inspired medical students to seriously consider a life filled with science; the scientist and mentor who stimulated his graduate students and junior investigators to tackle scientific problems of substance.

**Figure:**
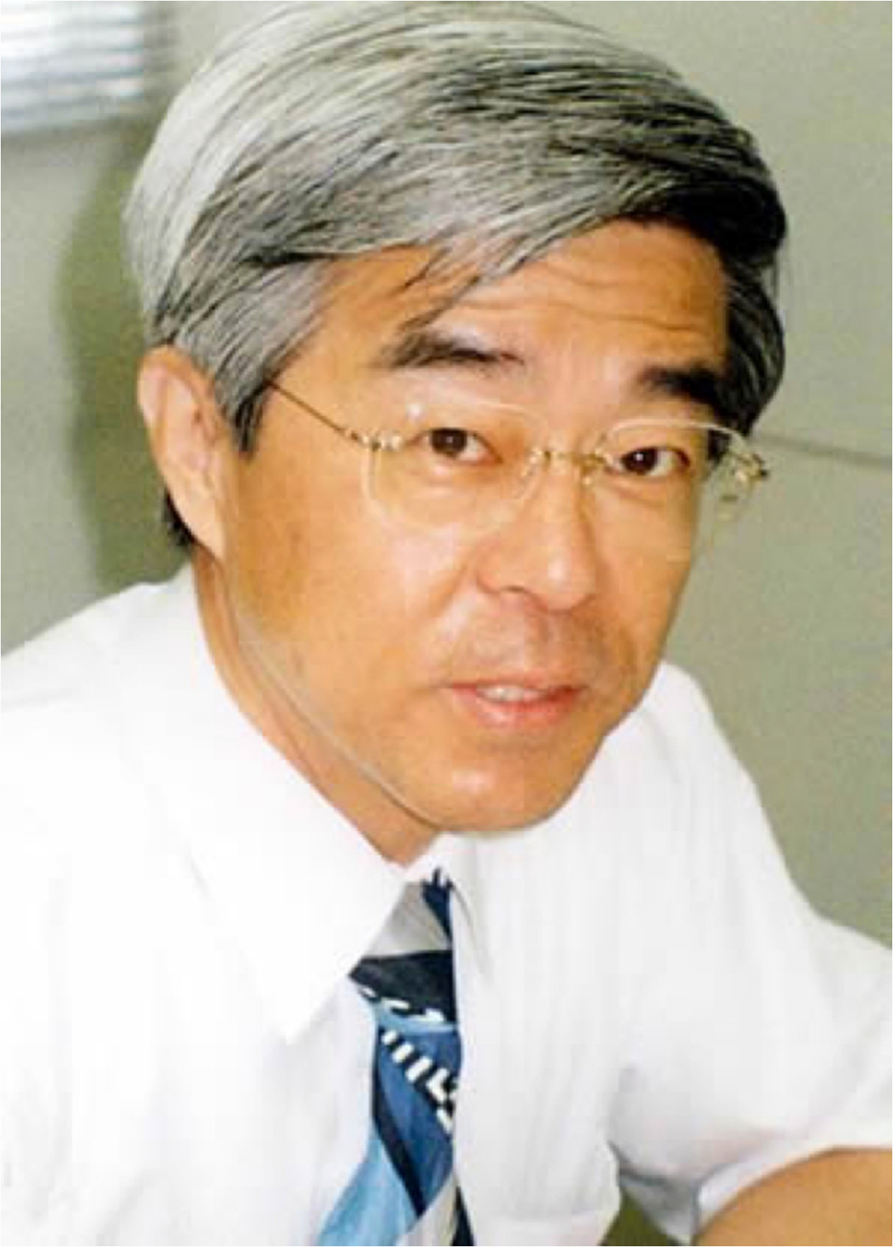
Dr. Yoshifumi Ninomiya.

This is the Yoshi that those who met and knew him will remember, but for me there is more. In 1980 he traveled from Japan to the United States with his family and arrived at Rutgers Medical School in New Jersey as my first postdoctoral fellow. He had received his MD degree and completed his residency at Okayama University Medical School, and he had graduated from the Tokyo Medical and Dental University PhD program. Dr. Yutaka Nagai had been his advisor so I took it for granted that the new postdoctoral fellow would be well trained in collagen biochemistry. What I had not foreseen was that Yoshi’s presence in my laboratory would turn out to be a life-changing event that established strong bonds between our families and profoundly affected our research directions and professional careers. It started an exciting journey that took us through discoveries of non-fibrillar collagens, changing our view of the roles of collagenous proteins in extracellular matrix architecture, and led us from matrix biochemistry to cell and developmental biology and genetics after we moved to Harvard Medical School in 1985.

Remarkably, our journey together did not come to an end when Yoshi left his Associate Professorship at Harvard and returned to Okayama University Medical School as Professor of Molecular Biology and Biochemistry in 1991. Our interactions continued through multiple meetings in Okayama while Yoshi built a strong department and put his stamp on extracellular matrix biology in Japan. Having one of his outstanding graduate students join my laboratory as a postdoctoral trainee also contributed to keeping our professional relationship strong. Finally, Yoshi started a very successful research internship program for medical students, and having 3^rd^ year students in the program join my laboratory for 3 months in the Fall semester became an annual reminder of Yoshi’s joyful approach to doing and teaching science.

I have often told my students and postdoctoral fellows that science is a marathon; not a sprint. Now the run of Yoshifumi Ninomiya, my collaborator and friend for the past 35 years, has come to an end. However, having seen the excited faces of Okayama students describing their research projects and sensed the pride and joy of Yoshi’s trainees presenting their findings, I know for sure that this marathon has not reached a finish line and that runners inspired by Yoshi will continue to run.

